# Squamous Cell Carcinoma of the Heel Invading the Calcaneus Treated by Radical Excision and Reverse Sural Flap

**DOI:** 10.7759/cureus.10740

**Published:** 2020-09-30

**Authors:** Oussama Mansour, Mohamad K Moussa, Ryan Bou Raad, Hussein Zreik, Ali H Allouch

**Affiliations:** 1 Orthopedics and Traumatology, Al Zahraa Hospital, University Medical Center, Beirut, LBN; 2 Orthopedic Surgery, Lebanese University Faculty of Medical Sciences, Beirut, LBN; 3 Hematology and Medical Oncology, Lebanese University Faculty of Medical Sciences, Beirut, LBN

**Keywords:** squamous cell carcinoma (scc), heel flaps, heel reconstruction, oncological surgery, fasciocutaneous flap, limb preserving surgery, heel cancer

## Abstract

Cutaneous squamous cell carcinoma (cSCC) is relatively rare in the heel and foot. It is characterized by great loco-regional aggressiveness but low metastatic potential. If left untreated, cSCC can grow to a large diameter. The rarity and unfamiliarity of this condition pose therapeutic difficulties to many surgeons. We hereby submit the case of a 32-year-old male patient presenting with a large 14 x 8 cm scaly, ulcerated, and bloody skin lesion covering the entire left heel and invading the calcaneus. The patient was treated with radical excision of the mass and a reverse sural fasciocutaneous flap to cover the remaining heel defect, with a very positive outcome and no complications.

This case is presented due to the rarity of the squamous cell carcinoma of the heel, and its large size (14 x 8 cm) in a relatively young patient, especially when it is invading the calcaneus.

## Introduction

Cutaneous squamous cell carcinoma (cSCC) is a common cancer arising from the malignant proliferation of epidermal keratinocytes [1]. It is the second-most common skin cancer; however, it is relatively rare in the foot, heel, and other non-sun-exposed areas [2].

Different risk factors are implicated in the development of cSCC. The most commonly recognized one is sunlight exposure, which plays a paramount role in fair-skinned individuals, where cSCCs most commonly arise in sites frequently exposed to the sun [3]. Carcinogenicity correlates with the chronicity of exposure. As a result, cSCC increases with age, particularly in sun-exposed areas [4]. The location of the cSCC is also affected by race; non-sun-exposed areas represent the most common location for cSCC in individuals with dark skin. In black individuals, common sites for cSCC include the lower legs, anogenital region, and areas of chronic inflammation or scarring [5]. Lesions that develop in relation to chronic scarring processes account for 20% to 40% of cSCCs in black patients [3].

Other risk factors include ionizing radiation, arsenic exposure, immunosuppression, family history of cSCC, and inherited disorders (xeroderma pigmentosum, epidermolysis bullosa, albinism, epidermodysplasia verruciformis) [6-7]. Chronic inflammation is also a risk factor of cSCC. Approximately 1% of cutaneous skin cancers arise in chronically inflamed skin (e.g., burn scars, chronic ulcers, sinus tracts, inflammatory dermatoses), and approximately 95% of these are cSCCs [8].

The occurrence of cSCC in the heel area represents a therapeutic challenge when planning for radical excision, especially when the calcaneus is locally invaded. Several heel reconstruction strategies are cited in the literature. We present herein a rare case of squamous cell carcinoma of large size (14 x 8 cm) invading the calcaneus in a young patient not having any risk factors. The patient was treated with radical excision and reverse sural flap.

## Case presentation

**Figure 1:**
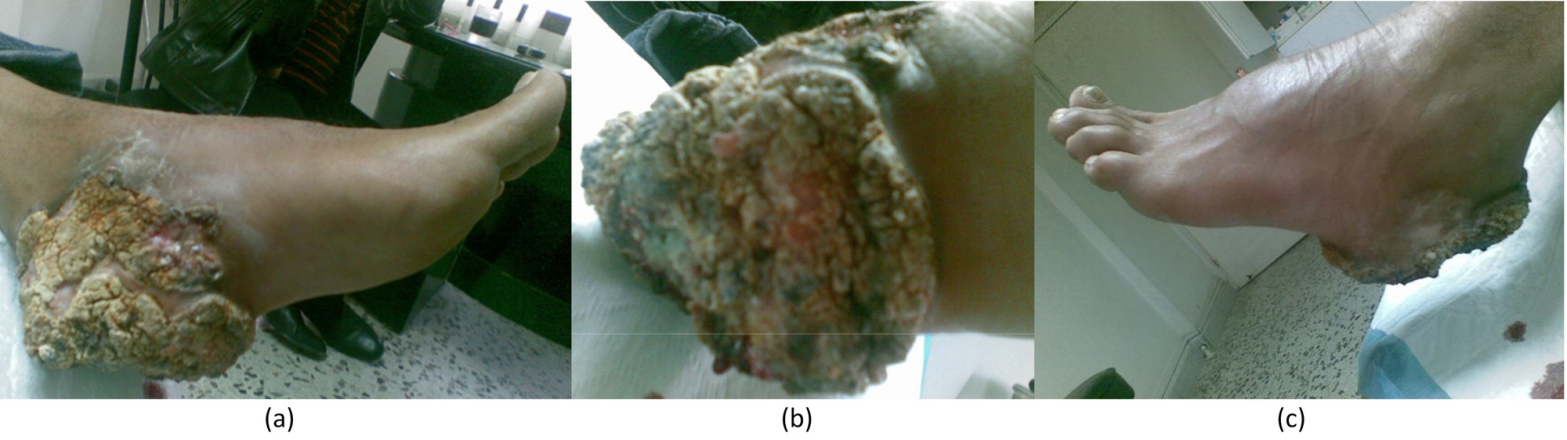
Inspection of the left lower limb showing a heel mass that is scaly, ulcerated, bloody, and covering the entire left heel with a size of 14 x 8 cm

This is a 32-year-old white Mediterranean patient who consulted our team for a lesion in the left heel. The patient reported a three-year history of a gradually growing mass on the left heel. He denies any history of burn, complicated wound, ulcer, or any chemical exposure to the heel. Upon physical examination, the mass was scaly, ulcerated, bloody, and covering the entire left heel, with a size of 14 x 8 cm (Figure 1). No regional lymph nodes were detected.

**Figure 2:**
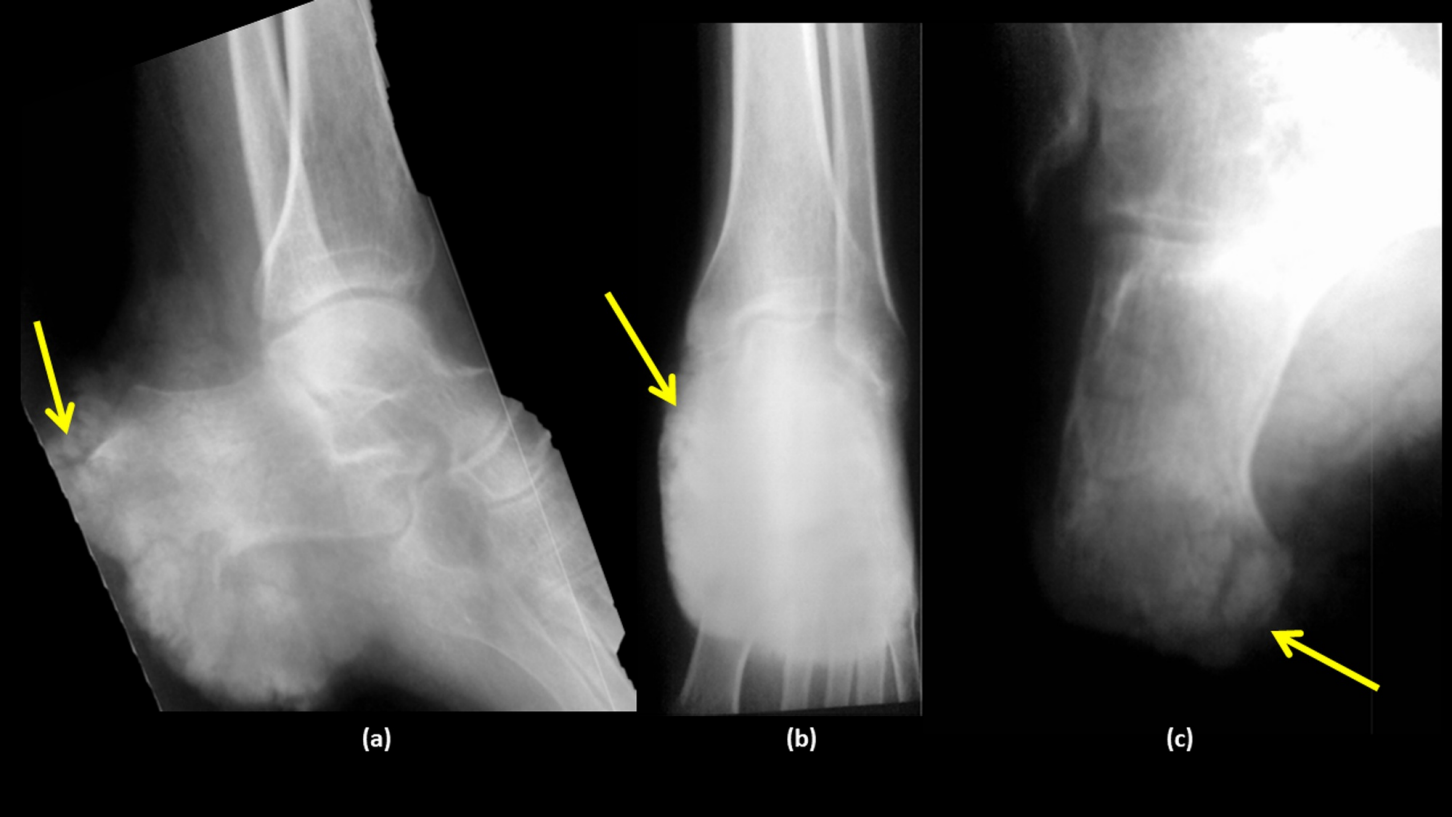
Radiographs of the left ankle showing a left-heel calcified mass with bony invasion (a) Lateral radiograph of the left ankle showing the involvement of the calcaneus, (b) AP radiograph of the left leg ankle showing the mass without the involvement of the tibiotalar joint, (c) calcaneal view showing the lytic lesion of the calcaneus AP: anteroposterior

Ankle radiographs were ordered and showed a left-heel calcified mass, with a bony invasion of the calcaneus (Figure 2). The workup was completed with a bone scan that confirmed the absence of any lesion outside the heel.

The patient was diagnosed with a large complex cutaneous tumor invading the calcaneus, and he was planned for surgical radial excision of the tumor and heel reconstruction surgery.

The patient was taken to the operating room and placed under general anesthesia in a prone position. The incision was made around the mass with 1 cm of skin margin. Dissection was carried out until reaching the calcaneus, where en bloc excision of the cutaneous mass was made, in addition to the macroscopically abnormal calcaneal bone, leaving in place the superior part of the calcaneal tuberosity that holds the Achilles tendon.

Afterward, a reverse sural flap was planned to cover the heel defect. The base of the flap was located 5 cm above the lateral malleolus. A reverse U-shaped incision was made on the posterior aspect of the calf. The fasciocutaneous flap was then mobilized. The cutaneous part of the flap that would be in contact with the defect was de-epithelialized.

The major part of the heel defect, which included the exposed bone, was covered using the mobilized flap. Subsequently, all remaining soft tissues at the recipient site were covered using full-thickness skin grafts taken from the lateral aspect of the thigh. The donor site was then covered with sterile dressing.

One month postoperatively, the recipient site was fully re-epithelialized. Therefore, the patient was taken to the operating room, and the flap was returned to cover the major part of the donor site. The remaining uncovered donor site was covered by a full-thickness skin graft.

**Figure 3:**
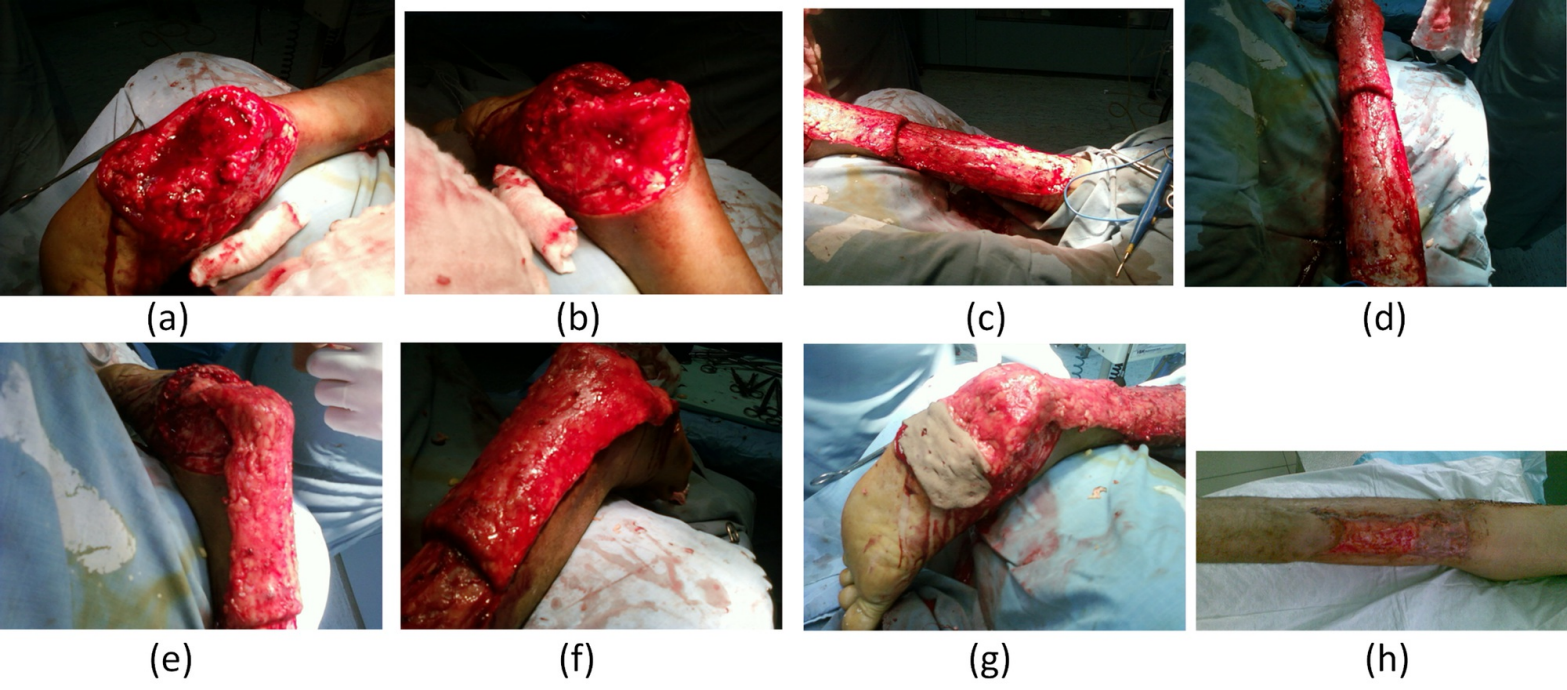
Important elements of the surgical interventions (a, b) Radical excision of the mass involving the calcaneus, (c, d) mobilization of the flap, (e, f) use of the flap to cover the heel defect, (g) all remaining soft tissues at the recipient site were covered using full-thickness skin grafts taken from the lateral aspect of the thigh (h) donor site at one month postoperatively was covered by a full-thickness skin graft

Figure 3 shows the most important elements of the surgical technique.

Pathology slides revealed squamous cell carcinoma of the skin, moderately differentiated, infiltrating the deep dermal tissue, the subcutaneous tissue, and the calcaneus, totally excised with a wide safety margin of resection

**Figure 4:**
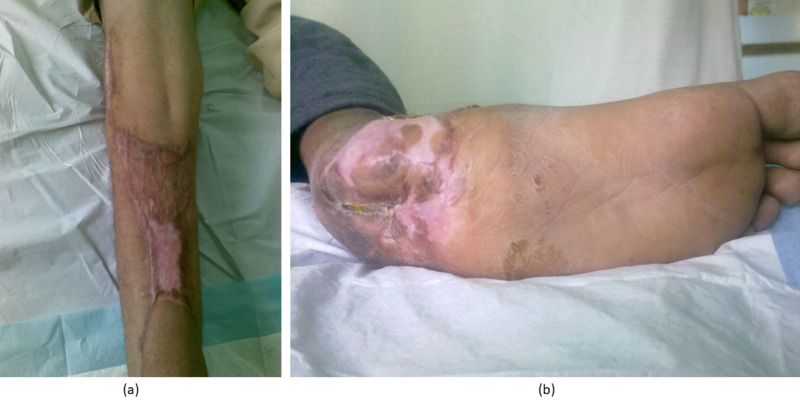
(a) Good skin condition at the donor site, (b) Good skin condition at the recipient site

Ten months postoperatively, the patient was free of signs or symptoms. The local skin condition was good at both the donor and recipient sites (Figure 4). The loss of heel height was compensated by a heel raiser.

## Discussion

cSCC can develop on any cutaneous surface, including the head, neck, trunk, extremities, oral mucosa, periungual skin, and anogenital areas [3]. However, its presence in the heel and foot is rare [2].

Several case reports of cSCC were published in the literature for cases that include an obvious risk factor for developing cSCC in the heel. Cavaliere R et al. reported a case of an elderly diabetic female with post-traumatic right heel non-healing pressure ulceration, which was found to be, in fact, cSCC (Marjolin's ulcer) [9]. In this case, the calcaneus was not exposed during surgical excision, which is why wound coverage was assured by vacuum-assisted closure. This option was not applicable in our case due to the size and depth of the cSCC in our patient where it invaded an important part of the calcaneus. Another risk factor was highlighted by Wukich DK et al. who reported a case of cSCC in a patient with longstanding chronic osteomyelitis [10]. Their patient has failed multiple reconstructive plastic and orthopedic procedures so he was treated by below-knee amputation. Furthermore, 10 cases of squamous cell carcinoma of the heel in patients previously affected by frostbite were reported by Rossis CG et al. [11], the biggest of which was an 8 x 8 cm macroscopically “fungating” tumor. These tumors were not invading the calcaneus and were all treated by excision and local flap followed by secondary excision in some cases of recurrence. Interestingly, unlike all these cases, our young patient is one of the very rare cases that does not dispose of any risk factors.

After diagnosis and workup, every patient should be stratified to determine if any high-risk features are present. Our patient fits in the category of local high-risk tumors taking into consideration its large size (14 x 8 cm), its deep invasion beyond subcutaneous fat to the bone, and its irregular borders [12]. Other high-risk features include recurrence, growth potential, degree of differentiation and invasion, site of prior radiotherapy or chronic inflammatory process, presence of neurological symptoms, and some high-risk subtypes such as acantholytic (adenoid), adenosquamous (showing mucin production), desmoplastic, or metaplastic/carcino-sarcomatous [13-14].

Primary treatment options and modalities for high-risk local cSCC include standard excision, using wide margins with a linear or delayed repair. If positive margins are not present after standard excision, patients should undergo second resection surgery, which, if failed, should be followed by multidisciplinary consultation to consider chemoradiation or clinical trial [15-16]. If negative margins are achieved after surgery with the presence of large nerve or extensive perineural involvement, adjuvant radiotherapy to the primary site is recommended [17].

Radical excision of cSCC of the heel is challenging, especially when the calcaneus is invaded. Being an area of relative hypovascularity and high weight-bearing requirement, the heel defect should be carefully addressed. Several heel reconstruction techniques have been described, including pedicled medial plantar flap, island medial plantar flap, reverse sural flap, free radial forearm flap, free rectus abdominis muscle flap, and free myocutaneous latissimus dorsi flap [18].

El-Shazly et al. developed, in 2008, a comprehensive algorithm for the reconstruction of a heel defect based on a retrospective cohort study. They found that the most suitable techniques for the treatment of a large defect in a setting of bone and Achilles tendon exposure are the reverse sural flap and the free flaps such as radial forearm flap, rectus abdominis flap, and latissimus dorsi flap [18]. As our patient fits in this category of recommendation and taking into consideration, the long operating time that requires this single-stage surgery of tumor radical excision followed by heel reconstruction, our decision was to dismiss the option of free flaps, which requires microvascular anastomosis. That is why we went for the easier, practical, and faster reverse sural flap, which gave us excellent wound coverage. Complications of the reverse sural flap, such as venous congestion, were not encountered in our case and the surgery was uneventful [19].

## Conclusions

cSCC of the heel is a rare medico-surgical condition that requires careful attention. Full workup and adequate preoperative planning are essential regarding tumor recurrence and heel complications. The decision about the reconstruction technique after radical resection should be based on the defect size, depth, involvement of the bone or tendons, and wound etiology (infection, trauma, post-cancer resection). The primary surgical goals should be focused on the excision of whole pathological tissues, conservation of the Achilles tendon function, and reconstruction of adequate heel contour. We elected to present one of the largest cSCCs of the heel invading the calcaneus due to the rarity of this condition in young patients not having any risk factors. Our patient was treated with radical excision of the tumor while conserving Achilles tendon insertion, and the heel was reconstructed using the reverse sural flap, which yielded positive outcomes.

## References

[1] Que SKT, Zwald FO, Schmults CD (2018). Cutaneous squamous cell carcinoma. Incidence, risk factors, diagnosis, and staging. J Am Acad Dermatol.

[2] Al Maksoud AM, Barsoum AK, Moneer M (2016). Squamous cell carcinoma of the heel with free latissimus dorsi myocutaneous flap reconstruction: case report and technical note. J Surg Case Rep.

[3] Gloster HM Jr, Neal K (2006). Skin cancer in skin of color. J Am Acad Dermatol.

[4] Juzeniene A, Grigalavicius M, Baturaite Z, Moan J (2014). Minimal and maximal incidence rates of skin cancer in Caucasians estimated by use of sigmoidal UV dose-incidence curves. Int J Hyg Environ Health.

[5] Mora RG, Perniciaro C (1981). Cancer of the skin in blacks. I. A review of 163 black patients with cutaneous squamous cell carcinoma. J Am Acad Dermatol.

[6] Kishikawa M, Koyama K, Iseki M (2005). Histologic characteristics of skin cancer in Hiroshima and Nagasaki: background incidence and radiation effects. Int J Cancer.

[7] Huang HW, Lee CH, Yu HS (2019). Arsenic-induced carcinogenesis and immune dysregulation. Int J Environ Res Public Health.

[8] Jellouli-Elloumi A, Kochbati L, Dhraief S, Ben Romdhane K, Maalej M (2003). Cancers sur cicatrice de brûlure: 62 cas (Cancers arising from burn scars: 62 cases) [Article in French]. Ann Dermatol Venereol.

[9] Cavaliere R, Mercado DM, Mani M (2018). Squamous cell carcinoma from Marjolin's ulcer of the foot in a diabetic patient: case study. J Foot Ankle Surg.

[10] Monaco SJ, Pearson K, Wukich DK (2015). Squamous cell carcinoma with chronic osteomyelitis: a case report. Foot Ankle Spec.

[11] Rossis CG, Yiacoumettis AM, Elemenoglou J (1982). Squamous cell carcinoma of the heel developing at site of previous frostbite. J R Soc Med.

[12] Schmults CD, Karia PS, Carter JB, Han J, Qureshi AA (2013). Factors predictive of recurrence and death from cutaneous squamous cell carcinoma: a 10-year, single-institution cohort study. JAMA Dermatol.

[13] Jambusaria-Pahlajani A, Kanetsky PA, Karia PS (2013). Evaluation of AJCC tumor staging for cutaneous squamous cell carcinoma and a proposed alternative tumor staging system. JAMA Dermatol.

[14] Karia PS, Jambusaria-Pahlajani A, Harrington DP, Murphy GF, Qureshi AA, Schmults CD (2014). Evaluation of American Joint Committee on Cancer, International Union Against Cancer, and Brigham and Women's Hospital tumor staging for cutaneous squamous cell carcinoma. J Clin Oncol.

[15] Lansbury L, Bath-Hextall F, Perkins W, Stanton W, Leonardi-Bee J (2013). Interventions for non-metastatic squamous cell carcinoma of the skin: systematic review and pooled analysis of observational studies. BMJ.

[16] Chren MM, Linos E, Torres JS, Stuart SE, Parvataneni R, Boscardin WJ (2013). Tumor recurrence 5 years after treatment of cutaneous basal cell carcinoma and squamous cell carcinoma. J Invest Dermatol.

[17] Varsha BK, Radhika MB, Makarla S, Kuriakose MA, Kiran GS, Padmalatha GV (2015). Perineural invasion in oral squamous cell carcinoma: case series and review of literature. J Oral Maxillofac Pathol.

[18] El-Shazly M, Yassin O, Kamal A, Makboul M, Gherardini G (2008). Soft tissue defects of the heel: a surgical reconstruction algorithm based on a retrospective cohort study. J Foot Ankle Surg.

[19] Ebrahimi A, Nejadsarvari N, Shams Koushki E (2012). Experience with reverse sural flap to cover defects of the lower leg and foot. Trauma Mon.

